# Endoscopic Transoral Resection of an Axial Chordoma: A Case Report

**DOI:** 10.5704/MOJ.1511.015

**Published:** 2015-11

**Authors:** S Taran, AH Yusof, MI Yusof

**Affiliations:** Department of Orthopaedics, Universiti Sains Malaysia, Kubang Kerian, Kelantan

**Keywords:** Cervical spine, endoscopic resection, transoral, chordoma

## Abstract

Upper cervical chordoma (UCC) is rare condition and poses unique challenges to surgeons. Even though transoral approach is commonly employed, a minimally invasive technique has not been established. We report a 44-year old Malay lady who presented with a 1 month history of insidious onset of progressive neck pain without neurological symptoms. She was diagnosed to have an axial (C2) chordoma. Intralesional resection of the tumour was performed transorally using the Destandau endoscopic system (Storz, Germany). Satisfactory intralesional excision of the tumour was achieved. She had a posterior fixation of C1-C4 prior to that. Her symptoms improved postoperatively and there were no complications noted. She underwent adjuvant radiotherapy to minimize local recurrence. Endoscopic excision of UCC via the transoral approach is a safe option as it provides an excellent magnified view and ease of resection while minimizing the operative morbidity.

## Introduction

Chordoma is a rare primary malignant tumour of notochordal origin. It is slow growing and locally invasive, however, may metastasize to other organs. The current treatment of choice for chordomas of the mobile spine and sacrum is en-bloc excision with wide margins and postoperative external-beam radiation therapy^1-3^. Only 6% of chordomas are localized at the cervical spine^3^. The resection of an upper cervical chordoma poses unique challenges to surgeons, hence, making wide resection surgery very challenging. Currently, transoral approach is the most widely performed technique to approach this area and has been accepted as the standard procedure among spine surgeons. To our knowledge, there is no report on the usage of Destandau endoscopic technique to excise an axial chordoma using the transoral approach.

## Case Report

**Fig. 1:**
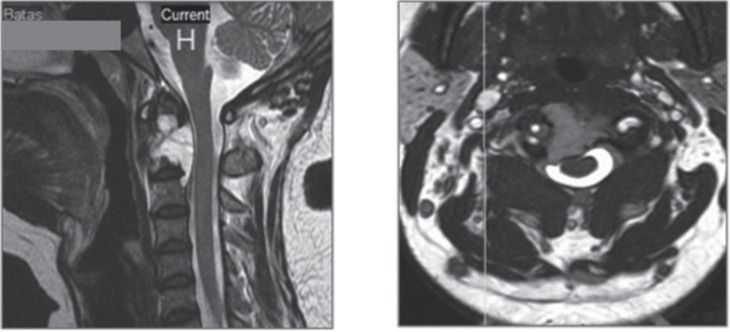
Pre-operative sagittal and axial views demonstrating the extent of the tumour on the cervical MRI.

We report a 44-year old Malay lady who presented to us with a 1 month history of insidious onset of progressive severe neck pain without neurological symptoms, dysphagia or evidence of metastasis. Her physical examination revealed hypertonia, hyperreflexia and a positive Hoffman’s sign for both upper limbs. Her erythrocyte sedimentation rate (ESR) on presentation was 100 mm/hr. Magnetic resonance imaging (MRI) revealed a lesion of the axis (2nd cervical vertebra C2) with extension superiorly along the dens and postero-inferiorly, as well as indentation of the spinal cord. The radiological features were suggestive of a chordoma (Figure 1).

She underwent a 2-stage surgical procedure. The first stage surgery was a posterior C1-C4 instrumented fusion (B. Braun Surgical system), while the second stage surgery was a transoral endoscopic excision of the axis, which was performed 3 months later.

**Fig. 2:**
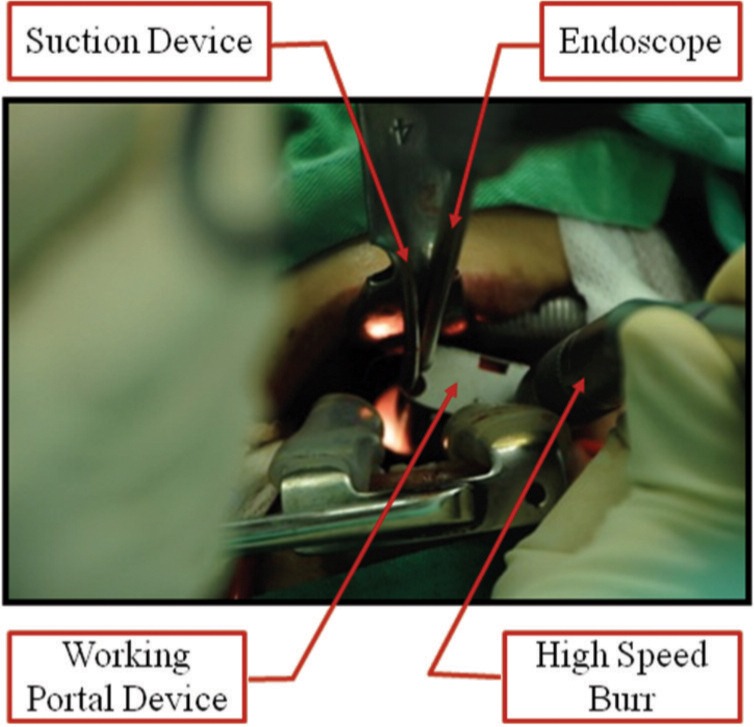
The mouth was maintained open using the Boyle-Davis mouth gag to enable the incorporation of the Destandau endoscopic system.

**Fig. 3:**
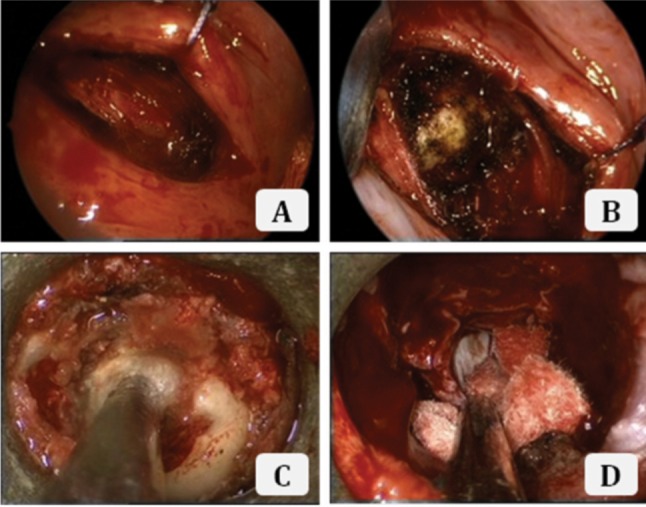
Endoscopic images: A. After incising the oropharangeal mucosa; B. After dissection to expose the axis; C. During high speed burring of the axial cortex; D. Revealing the dura after adequate piecemeal removal of the tumour.

**Fig. 4:**
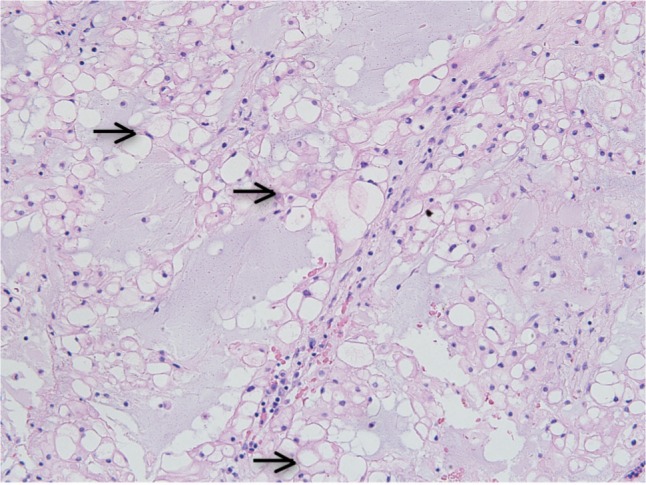
Photomicrograph demonstrating the chordoma cells with eccentrically located oval nuclei and vacuolated cytoplasm (physaliphorous cells) forming sheets that are embedded in mucoid stroma (H & E: 20X).

With regards to the operative technique for the second stage surgery, the patient was positioned in the supine position and orotracheal intubation was utilized for general anaesthesia, as shown in Figure 3. A Boyle-Davis mouth gag was employed to maintain the mouth open throughout the surgery (Figure 2) and the uvula was retracted using a soft rubber catheter trans-nasally. A needle and image intensifier was used to confirm the level of the anterior tubercle of the atlas, before a 3cm midline vertical incision was made on the posterior oropharyngeal mucosa and muscle (Figure 3A). Stay sutures were used to retract the incision edges. The anterior longitudinal ligament was exposed subperiosteally and the longus coli was mobilized laterally (Figure 3B). Next, the Destandau endoscopic system with the mobile Endospine® operating tube (Storz, Germany) was incorporated. A high-speed burr was introduced through the working portal of the operating tube and was used to remove the anterior cortex of the atlas and axis (Figure 3C). Intralesional excision of the tumour was performed entirely under endoscopic image guidance, using the burr and a bone punch, to achieve satisfactory anterior decompression (Figure 3D). The incision was closed using interrupted absorbable sutures. The blood loss was 350 ml and the duration of surgery was 9 hours. There were no immediate or delayed neurovascular complications, CSF leak or infection following the surgery. A 3-day prophylactic antibiotic course of intravenous cefuroxime and metronidazole was given in view of the long surgical duration, the usage of the oropharyngeal approach and the presence implants. The patient was able to swallow clear fluids by postoperative day 3. Histopathological examination of the bone and tissue samples taken intra-operatively confirmed the diagnosis of chordoma (Figure 4).

She underwent radiotherapy to the area of C1 – C3 nine months after the second stage surgery (45 Gys in 25 fractions over 5 weeks), in a different center due to logistic reasons. The patient is still under our follow up and 3 years post surgery, she is free from recurrence, metastasis and has no significant neurological deficit. Her screening was done using serial CT scans and physical examination.

## Discussion

Endoscopic excision of upper cervical chordoma using the Destandau endoscopic system through the transoral approach is an innovative and safe technique. Applying our experience of lumbar decompressive surgery using this system, we successfully performed excision of an upper cervical tumour.

In this case, the operative time was lengthy because it was our first time performing such a procedure. In addition, the tumour margin was wide and extra caution was needed. Nevertheless, the benefit of low blood loss and a magnified view throughout surgery is truly advantageous, especially in reducing complications such as cerebrospinal fluid leakage in the event of a dural injury.

Our decision to only perform intra-lesional excision to achieve satisfactory decompression was governed by the fact that a true en-bloc resection is not possible in most cases^4,5^. In the experience of Barrenechea *et al.*, 2007, piecemeal intra-lesional excision of cervical chordoma remains an effective technique when combined with post-operative radiotherapy^4^. The importance of adjuvant radiotherapy has also been stressed upon in literature, irrespective of the extent of the excision^2, 3, 5^.

In conclusion, though the technique involves a steep learning curve, endoscopic transoral approach is a safe surgical option to the upper cervical spine, besides providing an excellent magnified view and ease of resection. The Destandau endoscopic system is a versatile instrument that can be applied to many areas of the body. The horizon of its usage lies in the creativity of the surgeon to generate the best results.
